# The Hospital Officer's Bookshelf

**Published:** 1912-05-18

**Authors:** 


### THE HOSPITAL OFFICER'S BOOKSHELF.

A Manual of Surgical Treatment. By Sir W. Watson Cheyne, Bart., C.B., and F. F. Burghard, M.S., F.R.C.S. New edition, entirely revised with the assistance of T. P. Legg, M.S., F.R.C.S., and Arthur Edmunds, M.S., F.R.C.S. Vol. I. (Longmans, Green and Co. 1912. Pp. 552. Price 21s. net.)

This manual is one of those which long ago earned for itself the respect indicated by the bestowal of its authors' surnames as a soubriquet. "Cheyne and Burghard" is now twelve years old, or rather the first volume of it is; and not even the most inspired of text-books can reach that age without the need of drastic revision and re-arrangement; few are of much value in half that space of time. When younger authors take over the burden of work which such an enterprise entails, it is always a matter of interest to notice how far they can adapt themselves to the scheme of their predecessors, and how far their individualities change the previous scope and character of the book. In this case the original authors have not delegated the responsibility entirely to the juniors; and this no doubt accounts for the strong family resemblance between the revised and the older editions. In one important respect there is a distinct change, and a change for the better; this is in the general format and make-up, which are an improvement all round upon that of the original volumes.

The first of the series, which will consist of five volumes as before, contains the treatment of general surgical diseases, such as wounds, suppurations, infective diseases, tumours, and deformities. There is also an appendix upon anæsthetics and upon the examination of the blood. In dealing with inflammatory conditions and their treatment, the authors keep fairly close to the lines laid down twelve years ago in the first edition and sixteen years ago by Sir W. W. Cheyne in "Treves' System of Surgery." In a word, they are devoted adherents of the antiseptic rather than the aseptic school. On this topic they defend their views with much address and energy; and although a good many of the younger generation of surgeons will deride these views as behind the times, there is undoubtedly a lot of sound sense in the contentions of the four editors. Probably the course which commends itself to most practical surgeons in London just now is one midway between the exaggerated fear of carbolic and perchloride on the one hand and the too copious use of them in strong solutions on the other. We confess we cannot subscribe to all the teachings of Sir Watson Cheyne and his colleagues, especially about some of the uses of the "strong mixture," a fluid of terrific bactericidal and cellulicidal strength.

The essence of the work, as before, is always treatment; and no more is said upon diagnosis or ætiology than is necessary for elucidating the practical details of the measures recommended. The attention to every relevant detail is as careful as ever; and there are points of interest and originality to be found everywhere. The page dealing with the application of fomentations is an excellent specimen of this book at its best: if these directions were always followed to the letter, the results of treatment in septic lesions would probably be even better than they are. It is worth noting that the time-honoured custom of sprinkling (hot) laudanum on the surface of a newly wrung-out fomentation is advised for severe pain: we have long wondered whether this really does do any good, for it is hard to believe that the morphine can be absorbed through unbroken skin. Another old belief to which the authors subscribe is that an open fire in an operating-theatre leads to the decomposition into irritating vapours of any chloroform that is being administered to the patient. If this is the case, surely such dissociation can only occur right in the fireplace, where all detrimental products must immediately be withdrawn up the chimney by the draught.

Silk and catgut are the favourite suture materials of the authors. They may not have tried the now popular linen thread; but the merits of this material are so great that it is a pity no mention is made of it, especially as the second-rate suture of kangaroo tendon is described. Marine sponges, again, are preferred to dry swabs; here it is very doubtful if the authors will carry any considerable section of surgical opinion with them. It is also most regrettable that the photographs of a surgeon dressed for operating should show him with arms bare from the elbows to the edge of the gloves. Either stockingettes or long-sleeved gowns are surely *de rigueur* now in every clinic where serious attention is paid to Listerian principles. The same photographs show also the ordinary boots of the operator, and this is another small blemish to which exception must be taken.

The Fowler position is not mentioned in the section on the after-treatment of operations, which is far too much condensed altogether; and the treatment advised for so-called syncope arising during an operation is, in our judgment, thoroughly bad. The "anchor" stitch of Swan, Lynn Thomas, and others is not described; but otherwise the section on suturing methods is excellent. The chapters on various infective surgical diseases are very sound; they deal with principles very largely, for the local manifestations of tuberculosis and other infections are to be discussed in the regional sections of the work. The same holds good of tumours, which are classified in accordance with the most recent views, and only the general principles governing their extirpation are here discussed. Deformities are fully discussed, and various gymnastic and other methods of physical treatment receive the detailed exposition which is their due.

The appendix on anæsthetics is entrusted to the experienced pen of Dr. Silk, who is allotted only forty-seven pages for the elaboration of general, local, and spinal anæsthesia. It is clearly impossible that he can do it justice in this cramped space; but even allowing for that, there are several passages which ignore many of the most indisputable advances of the last few years. Dr. Emery, who contributes on the examination of the blood in surgical conditions, gives a clear and connected account of elementary first principles, but has no space for anything more than general remarks on the subject of vaccines. He might with profit have omitted the long account of the Thoma-Zeiss hæmocytometer, with which every medical student is now familiar.

In general terms it may justly be said, notwithstanding the above criticisms on points of detail, that the new edition of this excellent manual maintains, in its first volume at least, the very high tradition of practical value which the previous edition long ago acquired. It may be insular prejudice, but we have long cherished the notion that British text-books, taken all round, surpass very easily those produced in America and on the Continent. "Cheyne and Burghard" is a specimen of the high-class British text-book at its happiest and soundest; and if this edition enjoys as long a reign as the original, it will not be in the least surprising.

